# Analysis of hydroxocobalamin dosage in patients with CblC deficiency

**DOI:** 10.1186/s13023-025-03991-y

**Published:** 2025-08-21

**Authors:** Si Ding, Yuxin Deng, Yi Ding, Lili Hao, Wenjuan Qiu, Huiwen Zhang, Lili Liang, Ting Chen, Xia Zhan, Peng Xu, Chiju Yang, Hui Zou, Yongxing Chen, Shengnan Wu, Yufeng Wang, Min Yang, Xuefan Gu, Xianting Jiao, Lianshu Han

**Affiliations:** 1 Department of Pediatric Infection, Xinhua Hospital, Shanghai Jiao Tong University School of Medicine, 1665 KongJiang Road, Shanghai, 200092 China; 2 Department of Pediatric Endocrinology and Genetic Metabolism, Xinhua Hospital, Shanghai Institute of Pediatric Research, Shanghai Jiao Tong University School of Medicine, 1665 KongJiang Road, Shanghai, 200092 China; 3 Newborn Screening Center, Jining Maternal and Child Health Care Hospital, Jining, 272004 China; 4 Newborn Screening Center, Jinan Maternal and Child Health Care Hospital, Jinan, 250001 China; 5 Department of Endocrinology and Metabolism, Children’s Hospital Affiliated to Zhengzhou University, Henan Children’s Hospital, Zhengzhou, 450006 China; 6 Heze Maternity and Child Healthcare Hospital, Heze, 274032 China; 7 Department of Pediatrics, Shengjing Hospital of China Medical University, Shenyang, 110004 China

**Keywords:** Methylmalonic acidemia, *MMACHC* gene, Hydroxocobalamin, Dosage

## Abstract

**Objective:**

cblC deficiency is the most common organic acidemia in China. Hydroxocobalamin (OHCbl) is the main important therapeutic approach, while no approved protocols on its dosage during stable periods exist. This study aims to analyze OHCbl dosage and explore its influencing factors, providing reference for the option of OHCbl dosage.

**Methods:**

A total of 730 patients with cblC deficiency during stable periods were enrolled. Univariate analysis and multiple linear regression analysis were used to investigate the correlation between OHCbl dosage and tandem mass spectrometry (MS/MS)-based newborn screening (NBS), disease onset as well as *MMACHC* gene mutation.

**Results:**

Univariate analysis revealed no significant difference in OHCbl dosage between whether patients were diagnosed by MS/MS-based NBS or not, while significant differences were found based on disease onset and the presence of c.482G > A variant. Multiple linear regression analysis further identified disease onset and the c.482G > A variant as independent factors influencing OHCbl dosage. The median OHCbl dosage during stable periods was 1.18 mg/kg/week, with 0.31 mg/kg/week in patients with the c.482G > A variant and 1.37 mg/kg/week in those without. However, in patients carrying the c.482G > A variant, there was no significant difference in the OHCbl dosage between those with and without disease onset, while in patients without the c.482G > A variant, those with disease onset had a higher OHCbl dosage compared to those without.

**Conclusion:**

The study demonstrated the independent influencing factors of OHCbl dosage in patients with cblC deficiency and put forward corresponding reference for the option of OHCbl dosage.

**Supplementary Information:**

The online version contains supplementary material available at 10.1186/s13023-025-03991-y.

## Introduction

Combined methylmalonic acidemia and homocystinuria, cobalamin C type (cblC deficiency, OMIM 277,400), is the most common disorder of intracellular cobalamin (vitamin B12) metabolism, which is also the most common organic acidemia in China. The incidence of this disorder ranges from 1:37,000 to 1:200,000 worldwide [1–3] and varies between 1:3,500 and 1:39,000 in China [4, 5]. cblC deficiency has an autosomal recessive mode of inheritance and is caused by mutations in the *MMACHC* gene. This defect impairs the synthesis of adenosylcobalamin and methylcobalamin, essential coenzymes required for the conversion of methylmalonyl-CoA into succinyl-CoA (catalyzed by methylmalonyl-CoA mutase) and the remethylation of homocysteine (HCY) to methionine (catalyzed by methionine synthase), respectively. Characteristic metabolite biomarker abnormalities associated with this condition include elevated blood levels of propionylcarnitine (C3), C3 to acetylcarnitine (C2; C3/C2) ratio and HCY as well as urine methylmalonic acid and methylcitric acid, accompanied by normal or decreased methionine levels. These metabolic indicators form the primary basis for diagnosis and the assessment of the patient’s condition and prognosis. It was noted that optimal metabolic control and clinical response would occur when plasma HCY level was not exceeding 50µmol/L [6].

At present, the mainstay of therapy for cblC deficiency is intramuscular injections of hydroxylcobalamin (OHCbl), supplemented with oral drugs including betaine, L-carnitine and/or folic acid. Compared to the oral drugs with uncertain efficacy, OHCbl has been proven to be effective, safe and essential for the treatment of cblC deficiency [7, 8]. Guidelines by Huemer et al. [8] and Carrillo-Carrasco et al. [9] recommended an initial daily dose of 1 mg OHCbl, which then should be individually titrated based on metabolic response. For stable periods, published studies provide a wide range of OHCbl doses of 1-25 mg each time, once every 1–20 days [10–12] and these doses are rarely expressed in mg/kg/week, which puzzles the clinicians. The optimal dosages of OHCbl varied significantly among individuals with cblC deficiency has not been comprehensively explored [6]. It has been reported that patients’ prognosis improves with tandem mass spectrometry (MS/MS)-based newborn screening (NBS), which facilitates early diagnosis and treatment initiation. In addition, disease onset and genotype were also associated with outcomes [12–14]. However, the factors influencing the dosage of OHCbl remain unclear.

In this study, we investigated a large cohort of 730 Chinese patients with cblC deficiency who maintained HCY levels below 50 µmol/L during the follow-up. A detailed retrospective review of their clinical data was performed to analyze the dosage of OHCbl in patients during stable periods and to explore the influencing factors, providing clinical experience for practitioners.

## Methods

### Patients

A total of 730 patients with cblC deficiency were enrolled in this study, who were followed up at least once in Shanghai Xinhua Hospital from December 2006 to March 2024 and their plasma HCY levels were not exceeding 50µmol/L during the stable periods. While patients with worse metabolic control and clinical response were excluded. The clinical follow-up evaluation mainly included recent treatment strategies, condition changes, growth and development. In addition, blood levels of amino acids, acylcarnitines and HCY as well as urine levels of organic acids were analyzed regularly every 3–6 months. Written informed consent was obtained from the parents or legal guardians of the study participants. This study was approved by the Ethics Committee of Xinhua Hospital (approval No. XHEC-D-2024-064).

Patients who diagnosed by MS/MS-based NBS or not were included in the NBS and non-NBS groups, respectively. And patients with disease onset or not were divided into symptomatic and asymptomatic groups, respectively. Considering genotype-phenotype correlations, genotype-biochemical phenotype correlations and therapeutic effect [15, 16], patients were divided into five groups, including groups A, B, C, D and E. Patients in group A harbored homozygous or compound heterozygous variants of c.609G > A (p.W203X), c.658_660delAAG (p.K220del), c.567dupT(p.I190Yfs*13), c.656_658delAGA (p.Q219_K220delinsQ), c.217 C > T(p.R73X), c.315 C > G(p.Y105X) or c.445_446delTG(p.C149Hfs*32), all of which could cause a truncated or absent MMACHC protein and were reported to be related with early-onset cases [17, 18]. Patients in group B harbored homozygous or compound heterozygous variants of c.482G > A (p.R161Q), which is associated with late-onset cases and milder biochemical phenotypes [19, 20]. In addition, c.80 A > G (p.Q27R) and c.394 C > T (p.R132X), either homozygous or compound heterozygous, have been mainly reported in late-onset patients [11, 21]. Thus, patients in group C harbored homozygous variants of c.80 A > G or compound heterozygous variants of c.80 A > G and other variants except c.482G > A. Patients in group D harbored homozygous variants of c.394 C > T or compound heterozygous variants of c.394 C > T and other variants except c.482G > A and c.80 A > G. Patients in group E harbored other homozygous or compound heterozygous variants, which excludes the seven variants in group A as well as c.482G > A, c.80 A > G and c.394 C > T.

### Detection of metabolites

MS/MS was used to analyze blood levels of acylcarnitines, including C3, C2 and free carnitine as well as amino acids on dried blood filter papers by a tandem mass spectrometer (Applied Biosystems, API2000, API4000, API 4500, California, United State). C3/C2 ratios were calculated at the same time [22]. Gas chromatography-mass spectrometry (GC/MS) was used to detected urinary organic acids including methylmalonic acid and methylcitric acid by GC/MS (Shimadzu Limited, QP2010, QP2020, Kyoto, Japan) [23]. Plasma HCY were measured by fluorescence polarization immunoassay.

### *MMACHC* gene mutation analysis

Genomic DNA was extracted from peripheral blood samples. Gene test was performed by Sanger or next generation sequencing. Variations were identified by reference sequences from NCBI GENEBANK (*MMACHC*: NM_015506). The ClinVar database, the HGMD database, and the previous literatures were used to identify whether the Variations had been reported. The pathogenicity of novel variants was evaluated based on the American College of Medical Genetics and Genomics standards and guidelines [24].

### Treatment

After diagnosed, patients were administered with a vitamin B12 loading test, being treated with an intramuscular injection of OHCbl at a dose of 1-10 mg daily for three to five days based on age and weight, so as to reduce metabolites as far as possible. In the long-term treatment, as patients stabilized, the dosage was adjusted depending on the metabolic conditions of individual patients to leave plasma HCY levels within 50µmol/L [25]. In addition, oral administration of L-carnitine (50–100 mg/kg/day), betaine (50–100 mg/kg/day) and folic acid (5–10 mg/day) were also prescribed in most patients [7, 9].

### Statistical analysis

Statistical analyses were performed using SPSS 26.0 (IBM Corp., Armonk, New York) and SPSSAU (https://spssau.com/). Continuous variables were presented as the mean ± SD or median (interquartile range, IQR). Data that did not significantly deviate from normal distribution were tested using an unpaired two-tailed t-test, and non-normally distributed data were tested using the Mann-Whitney U test or Kruskal-Wallis test. Bonferroni’s correction was conducted to correct the bias of multiple testing. Multiple linear regression analysis was performed using variables with significant differences in univariate analysis and Box-Cox transformation was used to make the data of OHCbl dosage follow normal distribution. Statistical significance was established at *P*<0.05.

## Results

### Patient demographics

A total of 730 patients (median [IQR] age, 3.2 [1.3–6.1] years) were enrolled in this study, including 401 males and 329 females. Among them, 227 cases were asymptomatic and 503 cases had disease onset. Their symptoms mainly included feeding difficulty, vomiting, convulsion, lethargy, developmental delay, cognitive decline, motor involvement, psychiatric symptoms, renal dysfunction, pulmonary hypertension and anemia. A total of 390 patients were identified by MS/MS-based NBS, of whom 216 patients remained asymptomatic and 174 patients developed disease prior to treatment. Among 340 patients who did not experience MS/MS-based NBS, 329 cases were diagnosed symptomatically and 11 cases were confirmed via sibling diagnosis. The median follow-up time was 2 years and 3 months (range 3 months to 17 years). The levels of metabolite biomarkers (C3, C3/C2, methylmalonic acid and HCY) before and after treatment are presented in Table S1, which indicates the significant biochemical improvement after treatment. In addition, all of the HCY levels were within 50µmol/L (Table S1), and patients were treated with OHCbl at a dose of 1.5–45 mg each time, once a day to once a month according to their individuals’ condition. The median weight and dosage of OHCbl was 15 kg (IQR:10.5–22.0 kg) and 1.18 mg/kg/week (IQR: 0.63–1.79 mg/kg/week), respectively.

### Molecular genetic analysis

All 730 patients were sequenced for the *MMACHC* gene. Overall, 727 patients harbored compound heterozygous or homozygous variants, two patients had three variants in the two alleles and one patient carried only one variant. In total, 87 different kinds of variations were detected (Table S2). The ten most common variants are c.609G > A, c.658_660delAAG, c.482G > A, c.80 A > G, c.567dupT, c.656_658delAGA, c.394 C > T, c.217 C > T, c.315 C > G and c.445_446delTG, accounting for 85.08% and the remaining 77 variants accounted for 14.92%.

### Factors influencing the OHCbl dosage in patients with CblC deficiency

#### Univariate analysis

**MS/MS-based NBS** The OHCbl dosage and biochemical data at the last follow-up for each group of patients were summarized in Table 1. There were no significant differences in the dosage of OHCbl as well as levels of C3, C3/C2 radio, methionine and methylmalonic acid between NBS and non-NBS groups (*P*>0.05). As a result, the OHCbl dosage was unlikely to be influenced by whether patients were diagnosed by MS/MS-based NBS.

**Table 1:** Comparison of OHCbl dosage and biochemical data at the last follow-up in patients diagnosed by MS/MS-based NBS or not [Median (IQR)]

	OHCbl dosage (mg/kg/week)	C3(µmol/L)		C3/C2	Methionine (µmol/L)	Methylmalonic acid (mmol/molCr)	HCY(µmol/L)
**Reference range**	-	0.40-4.00		0.03–0.20	9.00–45.00	0.00–4.00	<15.00
**NBS** **(n = 390)**	1.17(0.63–1.79)	2.68(1.89–3.73)		0.13(0.09–0.18)	17.58(14.05–23.01)	4.47(1.88–10.51)	27.31(19.75–35.83)
**Non-NBS (n = 340)**	1.22(0.58–1.86)	2.70(1.85–3.97)		0.14(0.10–0.18)	18.17(14.74–22.63)	4.25(1.60–7.89)	29.30(23.20-39.34)
**P value**	0.954	0.078		0.194	0.572	0.146	**0.002**

-, no data

**Disease onset** As is shown in Table 2, symptomatic patients showed significantly higher dosage of OHCbl and levels of C3, C3/C2 ratio and HCY compared to asymptomatic patients (*P*<0.05), but there were no significant difference in the levels of methionine and methylmalonic acid between two groups (*P*>0.05). Thus, asymptomatic patients appeared to have a better response to OHCbl and show an optimistic prognosis.

**Table 2:** Comparison of OHCbl dosage and biochemical data at the last follow-up in patients with disease onset or not [Median (IQR)]

	OHCbl dosage (mg/kg/week)	C3(µmol/L)	C3/C2	Methionine (µmol/L)	Methylmalonic acid (mmol/molCr)	HCY(µmol/L)
**Reference range**	-	0.40-4.00	0.03–0.20	9.00–45.00	0.00–4.00	<15.00
**Symptomatic (n = 503)**	1.31(0.75–1.94)	2.89(2.07–4.02)	0.14(0.01–0.18)	17.90(14.50-22.77)	4.68(1.89–8.77)	30.00(23.60–39.00)
**Asymptomatic (n = 227)**	0.93(0.39–1.52)	2.56(1.75–3.53)	0.12(0.88 − 0.18)	17.94(13.79–22.87)	3.90(1.52–9.69)	25.00(14.90–34.20)
**P value**	**<0.001**	**<0.001**	**0.025**	0.614	0.328	**<0.001**

-, no data

***MMACHC*** **gene mutation** The OHCbl dosage and biochemical data at the last follow-up for patients who carried different types of variants were summarized in Table 3. Except for methionine levels, dosage of OHCbl and levels of C3, C3/C2 ratio, methylmalonic acid and HCY varied significantly among groups (*P*<0.05). Further, Bonferroni’s correction was applied. As a result, patients in group B showed significantly lower OHCbl dosage and C3 levels when compared to patients in other groups (*P*<0.05), while no significant differences were found among group A, C, D and E(*P*>0.05). Patients in group B showed significantly lower C3/C2 ratios compared to groups A and C (*P*<0.05), with no significant differences among other groups (*P*>0.05). Patients in group B also demonstrated significantly lower levels of methylmalonic acid compared to patients in group A, while there were no significant differences in the level of methylmalonic acid among the other groups (*P*>0.05). Additionally, patients in group B had significantly lower HCY levels than groups A, C, D and E (*P*<0.05), while group C showed significantly HCY levels when compared to group A, with no significant differences in HCY levels among other groups (*P*>0.05). These results illustrate that patients carrying the c.482G > A variant have a better metabolic control at a lower dosage of OHCbl than those who without this variant, while patients with other *MMACHC* variants show no significant difference in the dosage of OHCbl.

**Table 3:** Comparison of OHCbl dosage and biochemical data at the last follow-up in patients with different *MMACHC* gene mutation [Median (IQR)]

Categories	*N*	OHCbl dosage (mg/kg/week)	C3(µmol/L)	C3/C2	Methionine (µmol/L)	Methylmalonic acid (mmol/molCr)	HCY(µmol/L)
**Reference range**		-	0.40-4.00	0.03–0.20	9.00–45.00	0.00–4.00	<15.00
**Group A**	293	1.45(1.02–1.97)^a^	2.97(2.13-4.00)^a^	0.14(0.10–0.19)^a^	17.16(14.42–23.21)	5.20 (2.54–10.02)^a^	32.62(26.00-39.73)^a^
**Group B**	127	0.31(0.20–0.50)^b^	2.04(1.55–2.92)^b^	0.11(0.07–0.16)^b^	18.47(14.68–22.64)	2.38(0.84–7.90)^b^	16.00(12.00-26.27)^b^
**Group C**	115	1.33 (1.02–1.94)^a^	2.96(2.10–4.09)^a^	0.14(0.09–0.19)^ac^	19.44(14.33–23.71)	4.21(1.56–7.10)^ab^	28.00(22.40–36.40)^c^
**Group D**	36	1.24(0.81–1.79)^a^	2.89(2.14–3.94)^a^	0.14(0.10–0.16)^ab^	20.58(15.55–29.31)	4.10(1.67–8.50)^ab^	26.77(20.28–36.26)^ac^
**Group E**	159	1.17(0.65–2.06)^a^	2.88(1.95–4.11)^a^	0.14(0.10–0.18)^ab^	16.84(13.73–21.44)	4.10(1.60-11.01)^ab^	29.40(23.00–39.00)^ac^
**P value**		**<0.001**	**<0.001**	**0.004**	**0.149**	**0.002**	**<0.001**

Patients in group A harbored homozygous or compound heterozygous variants of c.609G > A, c.658_660delAAG, c.567dupT, c.656_658delAGA, c.217 C > T, c.315 C > Gor c.445_446delTG. Patients in group B harbored homozygous or compound heterozygous variants of c.482G > A. Patients in group C harbored homozygous variants of c.80 A > G or compound heterozygous variants of c.80 A > G and non-c.482G > A. Patients in group D harbored homozygous variants of c.394 C > Tor compound heterozygous variants of c.394 C > T and other variants except c.482G > A and c.80 A > G. Patients in group E harbored other homozygous or compound heterozygous variants, which excludes the seven variants in group A as well as c.482G > A, c.80 A > G and c.394 C > T

Columns with the same letters are not significantly different (*P*>0.05) and different letters indicate values are significantly different (*P* < 0.05)

-, no data

#### Multiple linear regression analysis

Multiple linear regression analysis was performed using disease onset and carrying the c.482G > A variant as independent variables and the OHCbl dosage as the dependent variable. The stepwise method was employed, with a significance level of P value <0.05 for variable selection, yielding an R value of 0.591 and the adjusted R-squared value of 0.348. The findings of the multivariate linear regression analysis were shown in Table 4. As a result, disease onset and carrying the c.482G > A variant are the independent factors of OHCbl dosage, and disease onset was positively associated with OHCbl dosage while carrying the c.482G > A variant was negatively associated with OHCbl dosage.

**Table 4:** Results of multiple linear regression analysis

Variable	Regression coefficient B	Standard error	Standardized regression coefficient	t value	*P* value	Variance inflation factor
**Constant term**	0.238	0.048		4.761	<0.001	-
**Disease onset**	0.127	0.054	0.073	2.370	0.018	1.060
**Carrying the c.482G > A variant**	-1.210	0.065	-0.570	-18.494	< 0.001	1.060

-, no data

#### Analysis of OHCbl dosage in patients with CblC deficiency

The median dosage of OHCbl was 1.18 mg/kg/week (IQR: 0.63–1.79 mg/kg/week) when 730 patients with cblC deficiency obtained a satisfactory metabolic control (HCY ≤ 50µmol/L) in this study. Given that disease onset and carrying the c.482G > A variant are the independent influencing factors of OHCbl dosage, we further explored the proper dosage of OHCbl in each patient based on the above two factors. As is shown in Table 5, a total of 127 patients carried the c.482G > A variant and their median dosage of OHCbl was 0.31 mg/kg/week (IQR: 0.20–0.50 mg/kg/week). There were no significant difference in the dosage of OHCbl between patients with disease onset or not(*P*>0.05). In 603 patients who did not harbor the c.482G > A variant, the median dosage of OHCbl was 1.37 mg/kg/week (IQR: 0.93-1.94 mg/kg/week). Among them, patients with disease onset showed higher dosage of OHCbl than those without (*P*<0.05).

**Table 5:** Analysis of OHCbl dosage in patients with CblC deficiency [Median (IQR)]

Categories	Disease onset	*N*	OHCbl dosage(mg/kg/week)	*P* value
**c.482G > A group (n = 127)**	Yes	57	0.32(0.20–0.49)	0.720
	No	70	0.30(0.20–0.50)	
**Non-c.482G > A group (n = 603)**	Yes	446	1.40(0.94–2.06)	**0.013**
	No	157	1.23(0.83–1.79)	

## Discussion

cblC deficiency is the most common type of methylmalonic acidemia in China, which can be prevented and treated. Once diagnosed, life-long treatment is required. If treatment is delayed, the rate of mortality and disability would be higher. Cobalamin is the cofactor related to methylmalonyl-CoA and homocysteine metabolism. Patients with cblC deficiency respond to parenteral cobalamin, preferably in the form of OHCbl, leading a considerable improvement in clinical manifestations and biochemical parameters [8, 9]. Vitamin B12 loading test is used to assess the efficacy of Vitamin B12 and reduce the levels of methylmalonic acid and HCY as soon as possible, with 1-10 mg of initial parenteral OHCbl daily for five consecutive days [8, 26, 27]. As patients stabilize, the frequency or dosage is decreased properly. However, limited research is available in the OHCbl dosage of patients with cblC deficiency during stable periods and clinical treatment is mainly based on individual experience. This retrospective study, therefore, aimed to elucidate the clinical data of 730 patients with cblC deficiency and to investigate the OHCbl dosage and its influencing factors during stable periods.

The goals of management are lowering methylmalonic acid and HCY as soon as possible and normalize methionine [8]. Based on the literature and our clinical experience, patients could obtain a satisfactory metabolic control when plasma HCY level was not exceeding 50µmol/L, and the levels of C3, C3/C2 ratio, methylmalonic acid and methylcitric acid levels tend to be within the normal range. It had been reported that patients experienced dose-dependent improvements of biochemical markers, especially methylmalonic acid and plasma HCY, via escalation of OHCbl dosage [28]. However, patients with the level of HCY around 50µmol/L or less showed subtle changes [6]. In addition, factors such as discomfort of OHCbl intramuscular injection and treatment costs should also be considered during the treatment [25]. Thus, proper OHCbl dosage should be received on an individual basis to obtain a satisfactory metabolic control.

In this study, 730 patients with cblC deficiency remained stable, receiving OHCbl at a dose of 1.5-45 mg each time, once every 1–30 days, with the median OHCbl dosage 1.18 mg/kg/week (IQR: 0.63-1.79 mg/kg/week). This indicates that there are large individual differences in OHCbl dosage when plasma HCY is within the expected range in each patient. According to the results of multiple linear regression analysis, disease onset and the presence of c.482G > A variant are the independent factors influencing OHCbl dosage. And the OHCbl dosage was positively correlated with disease onset, but negatively correlated with the presence of the c.482G > A variant.

The OHCbl dosage and levels of C3 and HCY were higher in patients with disease onset than those without, suggesting that clinically unaffected patients could achieve the same or better metabolic control at a lower OHCbl dosage compared to clinically affected patients. This might be related to the milder degree of metabolic abnormalities in clinically unaffected patients. Although there was no statistical difference in the OHCbl dosage between NBS and non-NBS groups, there were 216 patients (55.4%) who did not develop the disease after being diagnosed by MS/MS-based NBS and receiving timely treatment. Also, the level of HCY was significantly lower in patients diagnosed by MS/MS-based NBS than those without. Thus, patients could benefit from MS/MS-based NBS, with the capability of early diagnosis and treatment [13, 29].

The *MMACHC* gene mainly includes missense mutations, nonsense mutations, deletion variations and frameshift mutations. Also, genotype-phenotype correlations of some mutations had been demonstrated, which might be related to differences in the stability of mRNA and residual function of protein. Mutations, including c.217 C > T, c.445_446delTG, c.567dupT, c.609G > A, c.658_660delAAG, c.656_658delAGA and c.445_446delTG were observed primarily in patients with early-onset [30, 31]. All of them could result in the production of truncated or absent protein, or the deletion of critical amino acids the impaired function, causing more severe presentations [17, 18]. While c.482G > A, c.80 A > G and c.394 C > T mutations were common in patients with late-onset cases [11, 21]. All of them are missense mutations, resulting in the residual production of protein, besides c.394 C > T, which is a nonsense mutation. Lerner-Elli et al. found that levels of MMACHC mRNA transcript in cell lines homozygous for c. 394 C > T mutation were higher than those in cell lines homozygous for early-onset mutations [15]. This might be due to lower degrees of nonsense-mediated mRNA decay that the c. 394 C > T mutation had experienced, thus partially mitigating the effect of the mutant allele on cell function and resulting in the production of a truncated protein product with residual function. In addition, individuals who compound heterozygotes for an early-onset mutation and a late-onset mutation tend to present with late-onset disease since the early-onset mutation might be consistently underexpressed when compared to the late-onset mutation [15]. Reports on genotype and OHCbl dosage correlations are few. In this study, patients carrying c.482G > A variant showed significantly lower OHCbl dosage and levels of C3 and HCY when compared to patients without this variant. Also, the C3/C2 radio and methylmalonic acid levels in patients carrying c.482G > A variant were significantly lower than those with early-onset variants (group A). These findings are consistent with previous reports, suggesting that patients carrying c.482G > A variant appear to have a better response to OHCbl, even at a lower dosage [32]. Possible reasons might be related to the higher expression and intermediate stability of the c.482G > A variant [20, 33]. It is notable that there were no statistical differences in the OHCbl dosage among patients carrying early-onset variants (group A), c.80 A > G variant (group C), c. 394 C > T variant (group D) and other variants (group E) under stable metabolic conditions. However, patients with the c.80 A > G variant (group C) showed lower levels of HCY when compared those carrying early-onset variants (group A). This might be due to the fewer peptide chain changes and impact on the function of proteins caused by missense mutations as compared to nonsense mutations, deletion mutations and frameshift mutations [16, 17].

In order to further explore the proper dosage of OHCbl in each patient with cblC deficiency when a satisfactory metabolic control is obtained. Two factors, including disease onset and whether the c.482G > A variant was carried, which were incorporated in the multiple linear regression analysis were analyzed in this paper. As a result, the OHCbl dosage in patients carrying the c.482G > A variant when a satisfactory metabolic control is 0.31 mg/kg/week, which was not affected by disease onset. The OHCbl dosage in patients without c.482G > A variant when a satisfactory metabolic control is 1.37 mg/kg/week, and patients with disease onset required higher dosage of OHCbl than those without. Notably, OHCbl dosage in different patients with cblC deficiency varied greatly when a satisfactory metabolic control was obtained in our study, thus, further adjustments should be made according to the clinical manifestations and biochemical metabolites of different patients in actual clinical application.

This study has several limitations. Firstly, since cblC deficiency is the disease involving intracellular cobalamin metabolism, OHCbl, as the etiologic therapy, is the leading and indispensable treatment for this disease. While the benefit of betaine, L-carnitine and folic acid supplementation is yet not clear, and side effects and poor compliance might also exist [8, 34]. Thus, our study mainly focused on the OHCbl dosage and placed less stress on effects of betaine, L-carnitine and folic acid and patient adherence to these drugs, which should be verified in the further study. Secondly, our study involved patients from different hospitals with varying degrees of follow-up, preventing a complete clinical assessment. A more comprehensive evaluation will be conducted by recalling participants for further monitoring. Last but not least, the adjusted R square in the multiple linear regression analysis was 0.348 in this study, suggesting that other factors might play roles in OHCbl dosage. Further study should be conducted to investigate the effects of polymorphism of other genes associated with cobalamin metabolism, as well as environmental, ethnic, dietary and individual background genetic variations on the OHCbl dosage [15].

## Conclusion

Disease onset and carrying the c.482G > A variant are independent factors of OHCbl dosage in patients with cblC deficiency and the dosage was positively associated with disease onset yet negatively associated with carrying the c.482G > A variant. When patients with cblC deficiency during stable periods, the median OHCbl dosage was 1.18 mg/kg/week, which was 0.31 mg/kg/week and 1.37 mg/kg/week, respectively in patients who carried the c.482G > A variant and not. While among patients who did not harbor the c.482G > A variant, the OHCbl dosage in symptomatic patients was higher than that in asymptomatic patients. Thus, clinical application of OHCbl dosage should be adjusted individually.

## Supplementary Information

Below is the link to the electronic supplementary material.

Supplementary Material 1

## Data Availability

All data generated or analyzed during this study are included in this published article and its supplementary information files.

## Abbreviations

cblC: Cobalamin C
OHCbl: Hydroxocobalamin
HCY: Homocysteine
C3: Propionylcarnitine
C2: Acetylcarnitine
C3/C2: Propionylcarnitine to acetylcarnitine ratio
GC/MS: Chromatography/mass spectrometry
MS/MS: Tandem mass spectrometry
NBS: Newborn screening
