# Supplementary material for: Analysis of hydroxocobalamin dosage in patients with CblC deficiency
Source: Orphanet J Rare Dis. 2025 Aug 21;20:448. doi: 10.1186/s13023-025-03991-y (PMC12369215; doi:10.1186/s13023-025-03991-y)
Supplement: Supplementary file 1 — Supplementary Material 1 [file 13023_2025_3991_MOESM1_ESM.docx]

Supplemental

**Table S1 The biochemical results before and after treatment in 730 patients[Median (IQR)]**

|  | **C3(μmol/L)** | **C3/C2** | **Methionine**  **(μmol/L)** | **Methylmalonic acid (mmol/molCr)** | **HCY(μmol/L)** |
| --- | --- | --- | --- | --- | --- |
| **Reference range** | 0.40-4.00 | 0.03-0.20 | 9.00-45.00 | 0.00-4.00 | ＜15.00 |
| **Before treatment** | 6.44(4.50-9.10) | 0.57(0.49-0.89) | 8.74(6.16-13.68) | 112.97 (44.68-258.08) | 101.00(69.90-159.32) |
| **After treatment** | 2.75(1.95-3.91) | 0.14(0.10-0.18) | 17.92(14.41-22.79) | 4.30(1.80-9.02) | 28.10(21.48-37.27) |
| **P value** | **＜0.001** | **＜0.001** | **＜0.001** | **＜0.001** | **＜0.001** |

**Table S2 Results of *MMACHC* gene mutation test in** **730 patients**

| No. | Exon | Nucleotide mutation | Amino acid alteration | Number of alleles | Number of homozygous cases | Number of heterozygous patients | Variation frequency |
| --- | --- | --- | --- | --- | --- | --- | --- |
| 1 | E4 | c.609G>A | p.W203X | 562 | 102 | 358 | 38.47% |
| 2 | E4 | c.658_660delAAG | p.K220del | 141 | 11 | 114 | 9.58% |
| 3 | E4 | c.482G>A | p.R161Q | 140 | 12 | 116 | 9.65% |
| 4 | E1 | c.80A>G | p.Q27R | 136 | 5 | 127 | 9.31% |
| 5 | E4 | c.567dupT | p.I190Yfs*13 | 92 | 0 | 92 | 6.30% |
| 6 | E4 | c.656_658delAGA | p.Q219_K220delinsQ | 49 | 4 | 41 | 3.35% |
| 7 | E3 | c.394C>T | p.R132X | 40 | 0 | 40 | 2.74% |
| 8 | E2 | c.217C>T | p.R73X | 37 | 0 | 37 | 2.53% |
| 9 | E3 | c.315C>G | (p.Y105X) | 23 | 0 | 23 | 1.57% |
| 10 | E4 | c.445_446delTG | p.C149fs*32 | 23 | 0 | 23 | 1.57% |
| 11 | E1 | c.1A>G | p.M1V | 22 | 0 | 22 | 1.51% |
| 12 | E4 | c.481C>T | p.R161X | 20 | 0 | 20 | 1.37% |
| 13 | E1 | Exon 1 del |  | 19 | 0 | 19 | 1.30% |
| 14 | E3 | c.365A>T | p.H122L | 14 | 0 | 14 | 0.96% |
| 15 | E3 | c.331C>T | p.R111X | 9 | 0 | 9 | 0.62% |
| 16 | E4 | c.615C>A | p.Y205X | 7 | 0 | 7 | 0.48% |
| 17 | E4 | c.445_446insA | p.C149* | 6 | 0 | 6 | 0.41% |
| 18 | E4 | c.626_627delTG | p.V209Dfs*36 | 6 | 0 | 6 | 0.41% |
| 19 | E3 | c.398_399delAA | p.Q133Rfs*5 | 5 | 0 | 5 | 0.34% |
| 20 | E4 | c.440_441delGT | p.C149Hfs*32 | 5 | 0 | 5 | 0.34% |
| 21 | IVS1 | c.81+1G＞A |  | 5 | 0 | 5 | 0.34% |
| 22 | E4 | c.599G>A | p.W200X | 5 | 0 | 5 | 0.34% |
| 23 | E4 | c.626dupT | p.T210Dfs*35 | 5 | 0 | 5 | 0.34% |
| 24 | E4 | c.452A>G | p.H151R | 4 | 0 | 4 | 0.27% |
| 25 | E2 | c.228_231delTGAC | p.D77Qfs*22 | 3 | 0 | 3 | 0.21% |
| 26 | E3 | c.395_397delGAC | p.R132del | 3 | 0 | 3 | 0.21% |
| 27 | E4 | c.438G>A | p.W146X | 3 | 0 | 3 | 0.21% |
| 28 | E4 | c.457C＞T | p.R153X | 3 | 0 | 3 | 0.21% |
| 29 | E4 | c.541G>T | p.D181Y | 3 | 0 | 3 | 0.21% |
| 30 | E4 | c.565C>T | p.R189C | 3 | 0 | 3 | 0.21% |
| 31 | E4 | c.566_567insT | p.R189Rfs*14 | 3 | 0 | 3 | 0.21% |
| 32 | E4 | c.616C>T | p.R206W | 3 | 0 | 3 | 0.21% |
| 33 | E2 | c.271dupA | p.R91Kfs*14 | 2 | 0 | 2 | 0.14% |
| 34 | E3 | c.277-3_303del |  | 2 | 0 | 2 | 0.14% |
| 35 | E3 | c.427C>T | p.Q143X | 2 | 0 | 2 | 0.14% |
| 36 | E4 | c.448_449delATinsCC | p.I150P | 2 | 0 | 2 | 0.14% |
| 37 | E4 | c.606_641delinsCTT | p.W203_R214delinsF | 2 | 0 | 2 | 0.14% |
| 38 | E4 | c.624_625delTG | p.V209fs | 2 | 0 | 2 | 0.14% |
| 39 | E4 | c.668T>C | p.F223S | 2 | 0 | 2 | 0.14% |
| 40 | E1 | c.35_58delTCGAGGACACGCTATGTCCTTTTG | p.I12_G20delinsS | 1 | 0 | 1 | 0.07% |
| 41 | E2 | c.46C＞T | p.R16X | 1 | 0 | 1 | 0.07% |
| 42 | E1 | c.57del | p.F19Lfs*57 | 1 | 0 | 1 | 0.07% |
| 43 | E1 | c.90G>A | p.W30X | 1 | 0 | 1 | 0.07% |
| 44 | E2 | c.105G>A | p.E35E | 1 | 0 | 1 | 0.07% |
| 45 | E3 | c.144C>G | p.Y48X | 1 | 0 | 1 | 0.07% |
| 46 | E2 | c.166_174delCCTGCCATG | p.P56_M58del | 1 | 0 | 1 | 0.07% |
| 47 | E2 | c.179A>G | p.D60G | 1 | 0 | 1 | 0.07% |
| 48 | E3 | c.194A>T | p.H65L | 1 | 0 | 1 | 0.07% |
| 49 | E2 | c.271A>T | p.R91X | 1 | 0 | 1 | 0.07% |
| 50 | E3 | c.279_276delAG | p.S93Pfs*11 | 1 | 0 | 1 | 0.07% |
| 51 | E3 | c.298G>T | p.E100X | 1 | 0 | 1 | 0.07% |
| 52 | E3 | c.321G＞A | p.V107V | 1 | 0 | 1 | 0.07% |
| 53 | E3 | c.328_331delAACC | p.N110Dfs*13 | 1 | 0 | 1 | 0.07% |
| 54 | E3 | c.354G>C | p.Q118H | 1 | 0 | 1 | 0.07% |
| 55 | E3 | c.374G>A | p.G218N | 1 | 0 | 1 | 0.07% |
| 56 | E3 | c.388_390del | p.Y130del | 1 | 0 | 1 | 0.07% |
| 57 | E3 | c.393_395delACG | p.132_132del | 1 | 0 | 1 | 0.07% |
| 58 | E3 | c.396dupT | p.R132fs | 1 | 0 | 1 | 0.07% |
| 59 | E3 | c.420G>A | p.W140* | 1 | 0 | 1 | 0.07% |
| 60 | E4 | c.428G>A | p.W143X | 1 | 0 | 1 | 0.07% |
| 61 | E4 | c.432_433insAT | p.S146Yfs*19 | 1 | 0 | 1 | 0.07% |
| 62 | E4 | c.434dupT | p.S146fs | 1 | 0 | 1 | 0.07% |
| 63 | E4 | c.441_442delTG | p.C149fs | 1 | 0 | 1 | 0.07% |
| 64 | E4 | c.453_455del | p.151_152del | 1 | 0 | 1 | 0.07% |
| 65 | E4 | c.467G＞A | p.G156D | 1 | 0 | 1 | 0.07% |
| 66 | E4 | c.483G＞A | p.W161X | 1 | 0 | 1 | 0.07% |
| 67 | E4 | c.484G>A | p.G162R | 1 | 0 | 1 | 0.07% |
| 68 | E4 | c.485_487del | p.162_163del | 1 | 0 | 1 | 0.07% |
| 69 | E4 | c.534delA | p.K178Nfs*32 | 1 | 0 | 1 | 0.07% |
| 70 | E4 | c.538_540delCAT | p.H180- | 1 | 0 | 1 | 0.07% |
| 71 | E4 | c.540_547delTGACTGTG | p.H108Hfs*6 | 1 | 0 | 1 | 0.07% |
| 72 | E4 | c.547delG | p.V183Vfs*27 | 1 | 0 | 1 | 0.07% |
| 73 | E4 | c.561_572del | p.D188_A191del | 1 | 0 | 1 | 0.07% |
| 74 | E4 | c.565C>A | p.R189C | 1 | 0 | 1 | 0.07% |
| 75 | E4 | c.566delinsGT | p.I190Yfs*13 | 1 | 0 | 1 | 0.07% |
| 76 | E4 | c.566G>A | p.R189H | 1 | 0 | 1 | 0.07% |
| 77 | E4 | c.567_568insCCTT | p.I190Yfs*14 | 1 | 0 | 1 | 0.07% |
| 78 | E4 | c.568delA | p.I190Sfs*20 | 1 | 0 | 1 | 0.07% |
| 79 | E4 | c.568insT | p.A191Rfs*12 | 1 | 0 | 1 | 0.07% |
| 80 | E4 | c.569T＞G | p.I190S | 1 | 0 | 1 | 0.07% |
| 81 | E4 | c.575T＞C | p.L192P | 1 | 0 | 1 | 0.07% |
| 82 | E5 | c.610G>A | p.W204X | 1 | 0 | 1 | 0.07% |
| 83 | E4 | c.634delC | p.Q212fs | 1 | 0 | 1 | 0.07% |
| 84 | E4 | c.637G>T | p.E213X | 1 | 0 | 1 | 0.07% |
| 85 | E4 | c.643T＞C | p.Y215H | 1 | 0 | 1 | 0.07% |
| 86 | E4 | c.645C＞A | p.Y215X | 1 | 0 | 1 | 0.07% |
| 87 | E4 | c.651del | p.Q218Sfs*27 | 1 | 0 | 1 | 0.07% |
